# Beta-Thalassemia Major and Female Fertility: The Role of Iron and Iron-Induced Oxidative Stress

**DOI:** 10.1155/2013/617204

**Published:** 2013-12-16

**Authors:** Paraskevi Roussou, Nikolaos J. Tsagarakis, Dimitrios Kountouras, Sarantis Livadas, Evanthia Diamanti-Kandarakis

**Affiliations:** Hematology Unit & Endocrine Unit, 3rd Department of Internal Medicine, Medical School, University of Athens, “Sotiria” General Hospital, 152 Mesogeion Avenue, 11527 Athens, Greece

## Abstract

Endocrine complications due to haemosiderosis are present in a significant number of patients with beta-thalassemia major (BTM) worldwide and often become barriers in their desire for parenthood. Thus, although spontaneous fertility can occur, the majority of females with BTM is infertile due to hypogonadotropic hypogonadism (HH) and need assisted reproductive techniques. Infertility in these women seems to be attributed to iron deposition and iron-induced oxidative stress (OS) in various endocrine organs, such as hypothalamus, pituitary, and female reproductive system, but also through the iron effect on other organs, such as liver and pancreas, contributing to the impaired metabolism of hormones and serum antioxidants. Nevertheless, the gonadal function of these patients is usually intact and fertility is usually retrievable. Meanwhile, a significant prooxidants/antioxidants imbalance with subsequent increased (OS) exists in patients with BTM, which is mainly caused by tissue injury due to overproduction of free radicals by secondary iron overload, but also due to alteration in serum trace elements and antioxidant enzymes. Not only using the appropriate antioxidants, essential trace elements, and minerals, but also regulating the advanced glycation end products, could probably reduce the extent of oxidative damage and related complications and retrieve BTM women's infertility.

## 1. Introduction

In beta-thalassemia major (BTM), iron overload is the joint outcome of multiple blood transfusions and an inappropriately increased iron absorption associated with ineffective erythropoiesis [1]. The outpouring of catabolic iron that exceeds the iron-carrying capacity of transferrin results in the emergence of nontransferrin-bound iron (NTBI), which catalyzes the formation of free radicals, resulting in oxidative stress (OS) and damage to mitochondria, lysosomes, lipid membranes, proteins, and DNA [1]. Thus, thalassemics are in a state of enhanced OS [2].

Meanwhile, recent advances in the management of BTM have significantly improved life expectancy and quality of life of BTM patients, with a consequent increase in their reproductive potential and desire to have children [3]. However, endocrine complications due to haemosiderosis are still present in a significant number of patients worldwide and often become a barrier in their desire for parenthood [4]. Female patients with BTM usually suffer from hypogonadotropic hypogonadism (HH) associated with amenorrhea, anovulation, and infertility, attributed to the iron effect on the pituitary gland as well as on the female reproductive system. Early recognition and prevention of the endocrine complications, by early and regular chelation therapy, are mandatory for the improvement of the quality of life of these patients [5]. Also, treatment with combination of antioxidants and iron chelators could probably neutralize the deleterious effects of reactive oxygen species (ROS) [6] and probably reverse endocrine complications, improving reproductive ability and fertility potential.

In this paper, the published data on fertility potential of females with BTM are reviewed, trying to further associate the determinant role of increased iron and iron-induced OS observed in these patients, with their respective reduced fertility potential. The utility of substantial and theoretical therapeutic strategies is also discussed, which could counteract iron-induced OS and preserve BTM females' fertility capacity.

## 2. BTM and Fertility: Up-to-Date

BTM is a severe, transfusion-dependent anemia that causes infertility mainly due to iron deposition to endocrine organs after overtransfusion [7]. Spontaneous fertility can occur in well-chelated and transfused patients, but the majority are infertile due to HH and need assisted reproductive techniques (ART). Safarinejad has previously evaluated the hypothalamic-pituitary-ovarian axis in female patients with BTM [8]. In the thalassemic group, the baseline and peak levels, after GnRH test, of luteinizing hormone (LH), follicle-stimulating hormone (FSH), and estradiol were significantly lower than those in the control group [8]. However, ovarian function has been proposed to be merely preserved in women suffering from primary or secondary amenorrhea (PA and SA, resp.), as they become able to conceive, following a closely monitored stimulation therapy [4]. Overall, the report of a large number of successful pregnancies so far is highly indicative of the relative safety of pregnancy in the iron-adjusted BTM woman [4]. The iron-induced effect seems to have a central role in the pathogenesis of the decreased reproductive capacity [9].

Ovulation induction with gonadotropin has been sufficiently studied [10]. Skordis et al. have previously estimated the frequency of fertility among 50 women with BTM, of which 7 had PA, 9 had SA, and 34 had normal menstrual function (NM) [11]. In all patients with PA and SA, the pregnancies were induced, while, in most patients with NM, pregnancies were achieved spontaneously [11]. Additionally, in a previously published study, Danesi et al. showed that, by proper pharmacological stimulation, the steroidogenic function of the gonads and even ovulation can be reinstated in hypogonadal thalassemic women [12]. More recently, Origa et al. suggested that, in women with HH, gonadal function is usually intact and fertility is usually retrievable [3]. In this study, 46 women with BTM (58 pregnancies) were included, while conception was achieved after gonadotrophin-induced ovulation in 33 of them [3]. Moreover, Bajoria et al. have recently supported the feasibility and safety of pregnancy in females with BTM, reporting their experience on pregnancy following ART in 11 women with BTM, who had HH with functionally intact ovaries [13]. However, successful pregnancy and fertility were also suggested to be feasible in BTM patients with diminished gonadal reserves [14]. The safety of pregnancy for both mothers with BTM and their babies, with proper care and guidance, has been repeatedly proposed by several studies, even in the case of intensive transfusion and chelation treatment [7, 15, 16]. Meanwhile, human GH as an adjunct to hMG and hCG seemed to be a sensible approach in the treatment of infertile homozygous BTM patients [17].

Based on the above, it seems that, irrespectively of the spontaneous or induced ovulation, the current evidence-based knowledge on the outcome of attempted pregnancies in BTM female patients is optimistic. Also, the infertility observed in this group of patients is probably mostly attributed to impaired hypothalamic-pituitary-ovarian axis and less to gonadal dysfunction. However, there are minimal data directly associating the role of iron, iron-induced OS, and antioxidant supplementation with the fertility potential of these patients.

## 3. Iron and Pathophysiology of BTM Infertility

**Figure 1:**
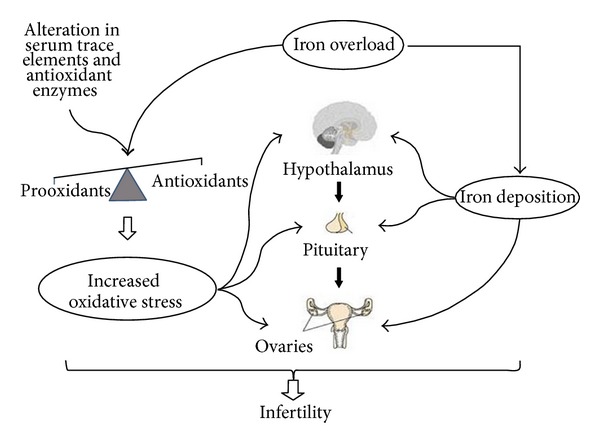
Infertility in female patients with BTM seems to be originated mainly by the direct and the indirect effect of iron overload. The combined effect of iron deposition and increased OS (because of a significant prooxidants/antioxidants imbalance) results in the dysfunction of the female reproductive axis.

Infertility in women with BTM is assumed to be mainly caused by the direct or indirect effect of iron on the hypothalamic-pituitary-ovarian axis and the female reproductive system. Indirect evidence suggests that the direct effect of iron is probably related to its direct deposition on the hypothalamic-pituitary axis and the female reproductive system, while direct evidence suggests that its indirect effect is mostly attributed to the iron-induced OS (Figure 1). However, there are limited data evaluating the pathophysiology of iron-induced compromised fertility, in which there is no clear discrimination between direct or indirect iron effects. Nevertheless, the most suitable model for speculating the direct and indirect effect of iron on BTM women's fertility capacity is the model of hemochromatosis, which will be further discussed below. Also, the iron-mediated effect on fertility could be discriminated in its hypothalamic-pituitary effect and in its effect on the female reproductive system.

### 3.1. Increased Oxidative Stress in BTM Patients

BTM patients are under continuous blood transfusion, which leads to an iron overload, with a resultant increase in NTBI that causes greater tissue toxicity than iron in other forms [2]. Another source of iron accumulation results from increased duodenal iron absorption due to decreased expression of hepcidin, the main regulator of iron homeostasis [6]. Iron generates production of ROS via the Fenton reaction; hence, iron overload disrupts the redox balance of the cells, causing chronic OS [18]. OS leads to lipid peroxidation of unsaturated fatty acids in membranes of cells and organelles. Cytotoxic byproducts of lipid peroxidation, such as malondialdehyde (MDA) and 4-hydroxy-2′-nonenal, are produced and these impair cellular function and protein synthesis and damage DNA [19]. Furthermore, a crucial component in the oxidant susceptibility of the thalassemic RBC is the release of heme and iron from the excessive, unpaired alpha-globin chains [2]. This release initiates self-amplifying redox reactions, which deplete the cellular reduction potential (e.g., glutathione/GSH), oxidize additional hemoglobin, and accelerate RBC destruction [2].

Oxidative/antioxidative balance is one of the most important factors for homeostasis. When the balance of ROS and antioxidants is disrupted towards an overabundance of ROS, OS occurs [20]. The generation of ROS is a steady-state cellular event in respiring cells, while their uncontrolled production often leads to damage of cellular macromolecules (DNA, protein, and lipids) and other small antioxidant molecules [21]. A number of major enzymatic and nonenzymatic defense mechanisms exist to neutralize and combat the damaging effects of these reactive substances [22]. The enzymic system functions by direct or sequential removal of ROS (superoxide dismutase/SOD, catalase/CAT, and glutathione peroxidase/GPx), thereby terminating their activities. Nonenzymic defense consists of scavenging molecules that are endogenously produced (GSH, ubiquinols, and uric acid) or derived from the diet, such as vitamins C and E [21]. These antioxidant nutrients occupy distinct cellular compartments and among them, there is active recycling. Antioxidative systems which protect from peroxidative damage are also supposed to be under hormonal influence, while antioxidative enzymes require the presence of microelements in their active centers as well as concert action of nonenzymatic antioxidants, which support enzymes in their scavenging action [23]. Meanwhile, diet seems to have an impact on antioxidative system, which requires appropriate supplementation in microelements and vitamins for its effective function, leading to scavenging excess of free radicals [23].

According to the current evidence-based knowledge, a significant prooxidants/antioxidants imbalance with subsequent increased OS exists in patients with BTM, which is mainly caused by tissue injury due to overproduction of free radicals by secondary iron overload, alteration in serum trace elements, and alteration in antioxidant enzymes level [24] (Figure 1). The results of Wassem et al. revealed that the levels of vitamin E and antioxidant enzymes GPx and SOD were significantly lowered in BTM patients, as compared with the control group, indicating that thalassemics are in a state of enhanced OS [2]. Another study revealed that plasma thiobarbituric acid (TBA) reactive substances were elevated in BTM children compared to controls, together with compensatory increase in SOD activity and decrease in CAT activity [25]. At the same study, serum ferritin showed a positive correlation with elevated TBA reactive substances and SOD, suggesting that iron overload is involved in the OS shown in cells [25]. A significant increase in the levels of lipid peroxide and iron and a significant decrease in the levels of vitamin E, total antioxidant capacity, and total iron binding capacity were observed in a different study [26], suggesting that OS and reduced antioxidant defense mechanism play an important role in pathogenesis of BTM [26]. Furthermore, Livrea et al. have previously suggested that the measurement of peroxidation products, matched with evaluation of antioxidants, may be a simple measure of iron toxicity in thalassemia, in addition to the conventional indices of iron status [27]. Their study showed that the mean concentrations of conjugated diene lipid hydroperoxides (CD), lipoperoxides evaluated as (MDA/TBA) adducts, and protein carbonyls increased about twofold with respect to control. A net drop in the concentration of ascorbate, vitamin E, vitamin A, beta-carotene, and lycopene was also observed. Serum levels of vitamin E were inversely correlated with ferritin, suggesting a major consumption of this antioxidant under iron overload, while serum levels of vitamin E, vitamin A and lycopene were inversely correlated with the levels of transminases [27]. On the other hand, their results pointed out that the iron-induced liver damage in thalassemia may play a major role in the depletion of lipid-soluble antioxidants [27]. Later on, Chiou et al. indicated that excessive lipid peroxidation and a profound depletion of plasma vitamins A, E, and C levels exist in patients with BTM, thus, antioxidant supplementation for the purpose of alleviating the OS may be warranted [28]. They also found that HCV infection did play some role in aggravating the depletion of plasma vitamins E and C levels in the BTM patients, while it did not seem to alter the levels of reduced GSH as well as antioxidant enzyme activities [28].

### 3.2. The Possible Impact of Oxidative Stress on BTM Females' Fertility Potential

Although there is no direct evidence-based publication, correlating the iron-induced OS with BTM women's infertility, existing published data on non-BTM patients only permit the correlation of OS (not specifically produced through iron overload) with normal and abnormal processes of the female reproductive system. Also, there is no available publication connecting iron-induced OS with specific altered signaling events in hypothalamic-pituitary-gonadal axis, which subsequently could lead to infertility. Thus, until now, only indirect evidence exists, which supports the hypothesis that iron-induced OS is the main causal agent for infertility in women with BTM. This is maybe happening not only through iron deposition and iron-induced OS in various endocrine organs, such as hypothalamus, pituitary and female reproductive system, but also through its indirect effect on other organs, such as liver and pancreas, contributing to the impaired metabolism of hormones and serum antioxidants.

Recently, ROS have been shown to have an important role in the normal functioning of reproductive system and in the pathogenesis of infertility in females [29]. OS develops when there is an imbalance between the generation of ROS and the scavenging capacity of antioxidants in the reproductive tract, while it affects both natural and assisted fertility [29]. ROS affect multiple physiological processes from oocyte maturation to fertilization, embryo development, and pregnancy [20]. There is sufficient evidence to hypothesize that dietary antioxidants and OS may influence the timing and maintenance of a viable pregnancy [30]. Also, physiological levels of ROS play an important regulatory role through various signaling transduction pathways in folliculogenesis, oocyte maturation, endometrial cycle, luteolysis, implantation, embryogenesis, and pregnancy [31]. Proper functioning of the ovary is critical to maintain fertility and overall health, and ovarian function depends on the maintenance and normal development of ovarian follicles [32]. The potential impact of OS on the wellbeing of primordial, growing and preovulatory follicles, as well as oocytes and early embryos, examining cell types and molecular targets, has been extensively reviewed [32]. Meanwhile, ROS-induced apoptotic cell death is involved in the mechanisms of corpus luteum (CL) regression that occurs at the end of the nonfertile cycle [33].

The role of OS in female reproduction is becoming increasingly important, as recent evidence suggests that it plays a part in conditions such as polycystic ovary syndrome (PCOS), endometriosis, unexplained fertility, spontaneous abortions, preeclampsia, hydatidiform mole, embryopathies, preterm labor, and intrauterine growth retardation [31]. Agarwal et al. have recently reviewed the role of OS in normal and abnormal female reproductive physiological processes, analyzing the exact mechanisms of redox cell signaling and the pathophysiology of OS-related reproductive diseases [34]. A special comment should be made on endometriosis patients, where iron overload has been demonstrated in the different compartments of the peritoneal cavity (peritoneal fluid, endometriotic lesions, peritoneum, and macrophages) [35]. This iron overload affects numerous mechanisms involved in endometriosis development [35]. Iron-induced OS may be involved in endometriosis-associated infertility and may play a role in the regulation of the expression of genes encoding immunoregulators, cytokines and cell adhesion molecules implicated in the pathogenesis of endometriosis [36]. Moreover, a recent study investigated the relationship between OS and the underlying causes of infertility, preovulatory ovarian hormones, and ovarian response to gonadotropin stimulation, in patients undergoing ART [37]. There was no significant relationship between plasma or follicular fluid (FF) total antioxidant capacity (TAC) and the underlying etiology of infertility [37]. However, there was a statistically significant positive association between FF E(2) levels and TAC. OS has an impact on the production of granulosa cell steroid hormones, in particular E(2), which is an important predictor of ovarian response [37]. Thus, the possible impaired ovarian response in BTM women could be probably caused by dysregulation in the production of granulosa cell steroid hormones, such as E(2). Meanwhile, it has been suggested that OS modulates the age-related decline in fertility [20]. Additionally, a recent study indicated that nonheme iron accumulation on ovarian stromal tissue may be related to aging of the ovary due to increasing OS [38]. The age-related decline in fertility due to OS has a special impact in our group of patients, because of their increased age average due to significant improvements in chelation treatment modalities. Furthermore, remarkable is another study which evaluated retinol and alpha-tocopherol, natural antioxidants that inhibit lipid peroxidation and protect against cell damage induced by OS, and revealed that lower concentrations of these natural antioxidants were associated with abnormal semen parameters in men and anovulation in women [39].

## 4. Evidence-Based Effect of Iron Overload and Iron-Induced OS on Hypothalamic-Pituitary-Gonadal Axis and Female Reproductive System

Although there are limited data directly correlating iron overload with iron-induced OS on BTM females' hypothalamic-pituitary-gonadal axis and ovarian reserve, indirect conclusions could be obtained through the model of hemochromatosis. 

### 4.1. The Model of Hemochromatosis

Endocrinopathies are common in transfusion-associated haemochromatosis [40], while HH is the most frequent endocrine abnormality in hemochromatosis [41]. The gonadotropin responsiveness to 100 micrograms of LHRH was impaired or absent in patients with hemochromatosis [42]. Also, in a cohort of 115 patients suffering from genetic hemochromatosis, hypogonadism was evidenced in 42% of them, in most of which it was considered as secondary to pituitary lesions as assessed by GnRH tests [43]. Moreover, in male patients with idiopathic hemochromatosis, testicular atrophy has been suggested to be caused by insufficient secretion of gonadotropins due to the selective accumulation of iron in gonadotropic cells of the pituitary gland [44]. Meanwhile, subnormal gonadotropin responses to GnRH, but normal ovarian reserve, as shown by normal follicular stimulation with hMG, were reported in a case of HH in a female with biopsy-proven hemochromatosis [45]. In another case report, recovery of reproductive functions, documented by hormone measurements, testicular biopsy, and semen analysis, was observed in a 37-year-old man with HH due to idiopathic hemochromatosis, after phlebotomy [46]. It has been also proposed that subjects with lesser degrees of hepatic siderosis at diagnosis are unlikely to develop hypogonadism [47]. Furthermore, the pituitary of a 69-year-old man with hemochromatosis had been previously removed at autopsy and was studied by histology, histochemistry, immunocytochemistry, electron microscopy, and X-ray diffraction [48]. Preferential localization of iron deposits was demonstrated in gonadotrophs, which, at the ultrastructural level, displayed selective, severe cellular injury [48]. X-ray diffraction revealed the deposition of iron-accumulated lysosomes, while iron storage was also noted in stellate cells [48]. It should be also noted that, in a group of 7 young male patients with genetic hemochromatosis, the normal or high increments of LH after LHRH stimulation suggested that secretion capacity of LH was intact and that hypothalamic dysfunction could be responsible for the observed preclinical gonadal deficiency [49]. A similar result was obtained in another male patient, where although the pituitary secretion of LH was normal in response to GnRH stimulation, clomiphene administration did not produce an increase in LH and FSH, suggesting that there was a defect in the hypothalamic GnRH response [50].

In agreement with the hemochromatosis model, abnormalities of the hypothalamic-pituitary-gonadal axis are the most common endocrine abnormalities in patients with BTM, as they require multiple blood transfusions leading to hemochromatosis [51]. Thus, based on the model of hemochromatosis that resembles iron-overloaded patients with BTM, it seems that abnormalities of ovulation and menstruation in these patients are most likely because of inadequate pituitary responsiveness to GnRH [45].

### 4.2. Direct and Indirect Evidence for the Hypothalamic-Pituitary and the Gonadal Effect of Iron

Patients with transfusional iron overload begin to develop pituitary iron overload in the first decade of life; however, clinically significant volume loss was not observed until the second decade of life [52]. Pituitary iron overload and volume loss were independently predictive of hypogonadism [52]. Pituitary R2 correlated significantly with serum ferritin as well as liver, pancreatic, and cardiac iron deposition by MRI [52]. Many patients with moderate-to-severe pituitary iron overload retained normal gland volume and function, representing a potential therapeutic window [52]. According to a different study, BTM patients with severe organ damage and iron overload are likely to be apulsatile with irreversible damage to their hypothalamic-pituitary axis, while those with less-severe iron overload are likely to have potentially reversible HH [53]. The same study suggested that gonadotrophin pulse parameters, rather than the gonadotrophin response to a GnRH bolus following prolonged pulsatile GnRH infusion, may be more useful in discriminating reversible from irreversible HH [53]. Furthermore, in a study of 33 patients above 15 years of age, with transfusion-dependent BTM and iron overload, anterior pituitary function (GnRH stimulation test) correlated well with MRI results. However, no correlation was found between the MRI measurements, the GnRH stimulation test, and the clinical status of the patients, as 28 out of the 33 patients achieved normal puberty [54]. Another possible mechanism of iron effect on hypothalamic-pituitary axis is through iron-induced OS, with subsequent alterations on elongation factor-2 (eEF-2) levels. The hypothalamic-pituitary system secretes peptide hormones, whose synthesis requires the integrity of the translation machinery. Among the possible causes of the decline of translation in old animals are the modifications of eEF-2 [55]. Iron-induced OS could be involved in the alterations of eEF-2, which forms adducts with MDA and 4-hydroxynonenal (HNE) [55]. The alterations of eEF-2 levels, secondary to lipid peroxidation and adduct formation with these aldehydes, could contribute to the suboptimal hormone production from these tissues [55]. Moreover, the possible concurrent GH deficiency may potentiate the already present OS, as patients with GH deficiency have an increased degree of OS and endothelial dysfunction, which possibly acts synergistically with iron-induced OS [56]. Thus, although there is limited evidence on the exact pathophysiological mechanisms of iron effect on hypothalamic-pituitary axis, it seems to be the basic pathogenetic cause of the impaired hypothalamic-pituitary system.

Meanwhile, according to our knowledge, only one study exists, which proves the iron deposition on the female reproductive system in patients with BTM. This particular study obtained histopathological evidence that deposition of haemosiderin occurred in the endometrial glandular epithelium of three patients with BTM [57]. This deposition was mainly evident in the apical part of these cells above the nuclei and should be taken into consideration as a contributing factor to the infertility in these patients, by altering endometrial receptivity for implantation [57]. In two patients who received effective iron chelating treatment with desferrioxamine, the endometrial haemosiderin deposits either disappeared or were significantly reduced [57].

### 4.3. Available Studies Directly Correlating Iron and Its Impact on Fertility in Women with BTM

The pathophysiology of iron-induced compromised fertility in women with BTM was only recently evaluated [9]. Low gonadotropin secretion resulted in reduced ovarian antral follicle count and ovarian volume, but levels of antimullerian hormone (AMH), a sensitive marker for ovarian reserve independent of gonadotropin effect, were mostly normal. AMH correlated with NTBI, suggesting a role of labile iron in the pathogenesis of decreased reproductive capacity, possibly occurring in parallel to cardiac iron toxicity, as cardiac iron was associated with the presence of amenorrhea and with NTBI levels. Thus, AMH was suggested to be valuable for future studies aiming at improved chelation for fertility preservation, whereas NTBI and labile plasma iron was suggested to be valuable for monitoring iron effect on the reproductive system [9]. The same research team explored the relationship between liver iron concentration (LIC) and fertility status in 26 females (mean 30 years old) with BTM. Seventeen (65%) of them experienced PA or SA. Levels of LH/FSH and estradiol were low or undetectable in 48% and 35% of patients, respectively, and did not correlate with age, presence of amenorrhea, and LIC. The fact that LH/FSH and estradiol, commonly used for assessment of fertility potential in thalassemia, had a poor predictive value, addressed the need for utilization of current available methods for assessment of fertility capacity in thalassemia [58].

## 5. Potential Antioxidants in BTM

Considering all the above, we suggest that the enhanced OS observed in iron-overloaded patients with BTM may play a significant role in their reduced reproductive ability. The investigation of the proper antioxidants for fertility and pregnancy in BTM female patients, which are recognized as patients of increased OS, is of great value and importance. The increased age average of this group of patients underlines the increased importance of OS on the female reproductive system, as it has been also connected with aging. However, despite the clear identification of the oxidative/antioxidative imbalance in patients with BTM, there are few studies evaluating the effect of different antioxidant supplementation in BTM-related morbidities, such as infertility.

### 5.1. Antioxidant Supplements Used in BTM

The benefits of vitamin C and vitamin E, as antioxidant supplements in BTM children, have been previously determined [59]. Twenty children who had laboratory confirmation of BTM at least 6 months with history of packed red cell transfusion without iron chelation were recruited. It was suggested that vitamin C plus vitamin E supplementations have benefits more than vitamin E alone in promoting antioxidant status and may enhance liver function, as total bilirubin tends to decrease [59]. Furthermore, curcuminoids, extracted from the spice turmeric, are known to have antioxidant and iron-chelating properties and have been proposed as a potential upstream therapy of thalassemia. Weeraphan et al. have recently applied proteomic techniques to study the protein profile and oxidative damage in the plasma of beta-thalassemia/Hb E patients before and after treatment with curcuminoids. Their study indicated the ameliorating role of curcuminoids towards OS and iron overload in the plasma proteome [60]. Glutamine, alpha-lipoic acid, acetyl-L-carnitine, and N-acetylcysteine are also recently being studied as antioxidant supplements for sickle cell disease and thalassemia [61]. Finally, the value of antioxidant supplements in the elimination of iron-induced OS and subsequent infertility issues could be speculated by the fact that various antioxidants (such as carnitine, vitamin C, vitamin E, selenium, carotenoids, glutathione, N-acetylcysteine, zinc, folic acid, and coenzyme Q10) are variably effective in OS-induced male-factor infertility, with respect to improving semen parameters and pregnancy rates [62].

### 5.2. Chelation Treatments as Antioxidants in BTM

Different studies have also focused on the antioxidant capacity of different chelation treatment modalities. Increased evidence from in vitro, in vivo, and clinical studies suggest that deferiprone (L1) can be used as a potent pharmaceutical antioxidant by mobilizing labile iron and copper and/or inhibiting their catalytic activity in the formation of free radicals and OS in tissue damage [63]. In contrast to L1, both desferrioxamine (DFO) and deferasirox (DFRA) were suggested to have major disadvantages in their use in noniron loading conditions due to toxicity implications [63]. Meanwhile, a subsequent study indicated that DFO chelation therapy does not normalize ferritin levels but attenuates oxidative damage and improves total antioxidant level in Malaysian Chinese BTM patients [64]. The objective of this research was to study the oxidant-antioxidant indices in BTM patients who were on desferrioxamine-chelation or without chelation therapy. Blood was collected from 39 Chinese patients and 20 controls. Plasma and peripheral blood mononuclear cell lysates (PBMC) were extracted and biochemical tests to evaluate OS were performed. OS was evident in these patients as advanced oxidized protein products (AOPP) and lipid hydroperoxides were elevated, whereas glutathione peroxidase activity and the ferric reducing antioxidant power (FRAP) were reduced. The catalase activity in the patients' PBMC was elevated, possibly as a compensatory mechanism for the reduced glutathione peroxidase activity in both red blood cells and PBMC. The lower FRAP and higher AOPP levels in the nonchelated patients compared with the chelated patients were indicative of a lower OS level in the chelated patients [64]. An additional study, which assessed whether oxidant-stress and inflammation in BTM could be controlled by DFRA as effectively as by DFO, revealed equal effectiveness in decreasing iron burden and levels of the oxidative-stress marker, MDA [65].

### 5.3. The Possible Effect of Advanced Glycation End Products (AGEs) Regulation on BTM Women's Ovarian Function and Fertility

Recent data have shown that OS is involved in the pathophysiology of anovulation [20, 66, 67]. While the role of OS in ovulatory dysfunction has been sufficiently studied [29, 68], advanced glycation end products (AGEs) have been recognized as mediators of increased OS [69, 70]. Advanced glycation end products (AGEs) are a heterogeneous group of bioactive molecules formed by the nonenzymatic glycation of proteins, lipids, and nucleic acids [71]. There is increasing evidence that AGEs play a pivotal role in atherosclerosis and in diabetes and its complications, while AGE accumulation is a measure of cumulative metabolic and OS and may so represent the “metabolic memory” [72]. Engagement of their receptor, RAGE, with AGEs is shown to activate its downstream signaling and evoke OS and inflammation in diabetes [73]. AGEs have been found to induce OS and conversely OS stimulates AGEs formation [74–76].

AGEs deposition in ovary dysregulate ovarian function, through the induction of androgen production, as well as OS generation, through the induction of NF-K*β* pathway, leading to increased production of atherogenic and inflammatory molecules. AGEs have been implicated in the development of insulin resistance, hyperandrogenism, and anovulation in polycystic ovary syndrome (PCOS) and seem to have a central role in PCOS pathophysiology, either as a marker of OS or through their specific actions after their ligation to their specific receptor RAGE [77]. Furthermore, there is substantial evidence that insulin resistance constitutes one of the main pathophysiological mechanisms leading to anovulation and ovarian hyperandrogenism in PCOS patients. Regarding the above, also that iron has been implicated in abnormal insulin secretion in patients with BTM or hemochromatosis [44, 78] and that OS might be responsible for a decline in insulin-mediated glucose uptake in BTM patients, leading to insulin resistance [79], we consider that the evaluation of AGEs, as markers of OS, in relation to iron overload, insulin resistance, and ovulation potential would be of great value. Reducing AGEs in BTM females could probably have beneficial effects in insulin resistance and ovulation potential, and thus in fertility potential. Finally, it is remarkable that the iron chelator Desferal had a retardation effect on the functional and structural changes of Hb during fructation [80]. It could prevent the AGEs and carbonyl formations and helix depletion during the Hb fructation process. Moreover, it could preserve peroxidase and esterase activities of fructated Hb similar to native Hb. Therefore, desferal could be introduced as an antiglycation drug to prevent the AGEs formation [80].

## 6. Conclusions

On the basis of all the presented data, it can be concluded that OS plays a major role in the pathophysiology of infertility in females with thalassemia. This OS is mainly caused by tissue injury due to overproduction of free radicals by secondary iron overload, alteration in serum trace elements, and alteration in antioxidant enzymes level. Consequently, there is a rationale for iron chelation to eliminate the free-iron species which, in this respect, act like antioxidants. Antioxidants are also capable of ameliorating increased OS parameters and, given together with iron chelators, may provide a substantial improvement in the pathophysiology of thalassemia [81]. However, there is no study evaluating the efficacy of antioxidant supplementation on BTM women's fertility potential. In agreement with previous suggestions, we consider that the application of treatment strategies that would reduce OS in the reproductive tract could help infertile women with diseases, such as BTM, that are caused by ROS/antioxidants imbalance [29]. In general, research is in progress to identify the mechanisms that are involved in the etiology of female reproductive diseases caused by ROS, and to create effective strategies that can counteract OS [29], while few are the trials investigating antioxidant supplementation in female reproduction [20]. The administration of selective antioxidants along with essential trace elements and minerals to reduce the extent of oxidative damage and related complications, such as infertility, in BTM patients, still needs further evaluation [24]. However, before clinicians recommend antioxidants, randomized controlled trials with sufficient power are necessary to prove the efficacy of antioxidant therapeutic strategies, in disorders of female reproduction [20], such as BTM. This is strongly emerged by the fact of prolonged survival and the newly recognized issues and challenges that adults with BTM face [82].
